# Cerebral endothelial cell derived small extracellular vesicles improve cognitive function in aged diabetic rats

**DOI:** 10.3389/fnagi.2022.926485

**Published:** 2022-07-14

**Authors:** Li Zhang, Chao Li, Rui Huang, Hua Teng, Yi Zhang, Min Zhou, Xiangshuang Liu, Baoyan Fan, Hao Luo, Annie He, Anna Zhao, Mei Lu, Michael Chopp, Zheng Gang Zhang

**Affiliations:** ^1^Department of Neurology, Henry Ford Hospital, Detroit, MI, United States; ^2^Department of Biostatistics and Research Epidemiology, Henry Ford Hospital, Detroit, MI, United States; ^3^Department of Physics, Oakland University, Rochester, MI, United States

**Keywords:** small extracellular vesicles, rat, neural stem cells, cerebral endothelial cell, cognitive function, diabete mellitus, exosomes

## Abstract

Small extracellular vesicles (sEVs) mediate cell-cell communication by transferring their cargo biological materials into recipient cells. Diabetes mellitus (DM) induces cerebral vascular dysfunction and neurogenesis impairment, which are associated with cognitive decline and an increased risk of developing dementia. Whether the sEVs are involved in DM-induced cerebral vascular disease, is unknown. Therefore, we studied sEVs derived from cerebral endothelial cells (CEC-sEVs) of aged DM rats (DM-CEC-sEVs) and found that DM-CEC-sEVs robustly inhibited neural stem cell (NSC) generation of new neuroblasts and damaged cerebral endothelial function. Treatment of aged DM-rats with CEC-sEVs derived from adult healthy normal rats (N-CEC-sEVs) ameliorated cognitive deficits and improved cerebral vascular function and enhanced neurogenesis. Intravenously administered N-CEC-sEVs crossed the blood brain barrier and were internalized by neural stem cells in the neurogenic region, which were associated with augmentation of miR-1 and –146a and reduction of myeloid differentiation primary response gene 88 and thrombospondin 1 proteins. In addition, uptake of N-CEC-sEVs by the recipient cells was mediated by clathrin and caveolin dependent endocytosis signaling pathways. The present study provides *ex vivo* and *in vivo* evidence that DM-CEC-sEVs induce cerebral vascular dysfunction and neurogenesis impairment and that N-CEC-sEVs have a therapeutic effect on improvement of cognitive function by ameliorating dysfunction of cerebral vessels and increasing neurogenesis in aged DM rats, respectively.

## Introduction

Diabetes mellitus (DM) is a common metabolic disease currently affecting more than 425 million people globally (Eid et al., 2019). DM accelerates cognitive impairment and increases the risk for dementia in the elderly (Biessels et al., 2006). Clinical trials show that even though medications that control blood glucose levels have greatly improved diabetes management, they do not prevent progression of cognitive declines (Peñaherrera-Oviedo et al., 2015; Simó et al., 2017). With increasing lifespan and rising prevalence of DM around world, there is a compelling need to develop new therapeutic strategies to improve cognitive functions in the elderly DM population (Barzilai et al., 2012).

Cerebral vascular function and neurogenesis are coupled and contribute to cognitive function in particular to learning and memory processes (van Praag et al., 2002; Deng et al., 2009). Neurogenesis has been demonstrated in the hippocampus of adult neurologically healthy human subjects, whereas patients with Alzheimer’s disease have substantial reduction of hippocampal neurogenesis (Spalding et al., 2013; Moreno-Jiménez et al., 2019). In rodents, DM induces cerebral vascular dysfunction and dramatically reduces adult neurogenesis (Stranahan et al., 2008; Takechi et al., 2017). Although multiple soluble factors released by cerebral endothelial cells (CECs), such as vascular endothelial growth factor, that have been shown to mediate neurogenesis (Shen et al., 2004), key molecules that coordinate the communication between CECs and neural stem cells (NSCs), particularly under DM condition, have not been fully elucidated.

Small extracellular vesicles (sEVs), exosomes, are endosomal origin membranous nanovesicles with a size of ∼40 to 150 nm in diameter and mediate intercellular communication by transferring their cargo proteins, lipids, and genomic materials including microRNAs (miRNAs) between source and recipient cells (Kourembanas, 2015). We have demonstrated that sEVs released by healthy CECs promote NSCs to differentiate into neurons, suggesting that sEVs are involved in coupling of cerebral endothelial cell and NSC function (Zhang et al., 2020). Moreover, sEV-based treatments reduce neurovascular injury and stimulate neurogenesis in various animal models of neurological diseases and injury including stroke, traumatic brain injury, Alzheimer’s disease, and Parkinson’s disease (Zhang and Chopp, 2016; Chen et al., 2020). Whether CEC derived sEVs (CEC-sEVs) mediate the communication between CECs and NSCs, and play a role in DM induced cognitive impairments has not been studied.

Experimental studies in aged DM animals are limited. One main reason may be that these diabetic animals suffer from short life span (∼ 8 to 14 months), which precludes their use for studying the effect of DM on vascular function and neurogenesis during aging and for investigating associated cognitive impairment (Azain et al., 2006; Sataranatarajan et al., 2016). Using nicotinamide (NTM) and streptozotocin (STZ) which partially damage insulin producing pancreatic β-cells, we have generated a model of DM in the rat at age 13 months (Zhang et al., 2016). These rats exhibit progressive cerebral vascular dysfunction, reduced neurogenesis and cognitive decline at age 15 to 17 months, which reflect many aspects observed in patients with type 2 DM (Masiello et al., 1998; Zhang et al., 2016). Using this rat model, we investigated the role of sEVs derived from CECs of DM rats (DM-CEC-sEVs) in mediating diabetes-induced neurovascular damage and impairments on neurogenesis. Additionally, we examined the therapeutic effect of sEVs derived from healthy CECs (N-CEC-sEVs) on amelioration of DM induced cognitive impairment. Here, we present the first evidence that DM-CEC-sEVs suppress NSC neurogenesis and exacerbate endothelial permeability, whereas N-CEC-sEVs have a therapeutic effect on improvement of cognitive function in aged DM rats.

## Materials and methods

All experimental procedures were approved by the Institution Animals Care and Use Committee of Henry Ford Hospital. All outcome assessments were performed by observers who were blinded to the treatments.

### Animal model

To mimic the general DM population, middle aged male Wistar rats (13 month of age) were subjected to intraperitoneal injection of NTM (210 mg/kg) and STZ (60 mg/kg). Non-fasting plasma glucose levels were measured 7 days after NTM/STZ injection and monthly thereafter using a portable blood glucose meter (AgaMatrix). Animals with glucose concentrations >200 mg/dL were enrolled. This model has been demonstrated to produce noninsulin-dependent DM syndromes that resemble human T2DM (Masiello et al., 1998). Aged-matched rats without NTM-STZ injection were used as controls.

### Primary cerebral endothelial cells culture, small extracellular vesicle isolation, and characterization

Primary CECs were isolated from cerebral microvessels of healthy male adult rats (2–3 months of age) and aged-DM rats (15 months of age, 2 months of DM) according to published method (Teng et al., 2008). We have demonstrated that the purity of primary endothelial cells is above 90% (Teng et al., 2008). The sEVs were isolated from culture medium of passages 1 to 2 CECs using a ultracentrifugation method according to published protocol (Li et al., 2021). The size and particle number of sEVs were characterized by a nanoparticle tracking analyzer (NTA, NanoSight N300 System, Merkel Technologies, Israel). sEV morphology was examined by means of transmission electron microscopy (TEM, JEM-1500Flash, JEOL). Total proteins extracted from isolated sEVs were used for Western blot analysis for the identification of EV marker proteins. The following primary antibodies were used: CD63 (ab68418, 1:1,000, Abcam), Alix (ab88388, 1:1,000, Abcam), CD31 (ab124432, 1:1,000, Abcam), and Calnexin (ab22595, 1:1,000, Abcam).

### Treatment of aged-diabetes mellitus rats with N-cerebral endothelial cells-small extracellular vesicles

To examine the therapeutic effects of N-CEC-sEVs, aged-DM rats at 2 months after NTM-STZ injection (15 months of age, *n* = 14/group) were randomly assigned to receive twice weekly intravenous administration of the N-CEC-sEVs at concentrations of 1 × 10^11^ particles/injection, or the same volume of Vehicle (saline) for a total of four consecutive weeks. For detecting cell proliferation, bromodeoxyuridine (BrdU, 100 mg/kg), a thymidine analog, was injected (i.p) daily for 7 days starting 2M after NTM-STZ injection. Rats were euthanized 4 months after NTM-STZ injection (17 months) and brain tissues were collected for immunohistochemistry and Western blot analysis.

### Behavioral tests

For evaluation of cognitive function, odor recognition test (Levy et al., 2004) and social interaction test (Sato et al., 2012) were performed at 2 and 4 months after DM onset. Morris water maze (MWM) test (Choi et al., 2006) was performed 4 months after DM onset (*n* = 10/group). These tests are well established in our laboratory and provide sensitive evaluation of cognitive function (Zhang et al., 2016).

The odor recognition test evaluates the non-spatial social olfactory memory that critically depends on adult neurogenesis (Levy et al., 2004). Briefly, rats were gradually introduced to four wooden beads consistent with two beads with its home cage odor (F1 and F2); one bead has a recently familiarized odor from a donor rat (N1), and one bead has a never encountered novel-odor from another donor rat (N2). The test was performed in three phases including a familiarization phase, an odor habituation phase, and an odor recognition memory test phase (Spinetta et al., 2008). During the initial familiarization phase, four unscented wooden beads (designated as F1–F4) were introduced into the home cage, where the testing rat would be familiarized with the presence of the beads for 24 h. The odor habituation phase of the task was done the next day after the now familiar beads with home cage odor (F1–F4) were removed for 1 h. The rat was exposed to four beads including three familiar beads (F1–F3) and a bead with odor from a donor rat (N1) for three 1-min trials with 1-min intertrial intervals. This procedure produces habituation to N1. The odor recognition memory test was conducted 24 h after the odor habituation phase. The testing rat was exposed to four beads including two with home cage odor (F1 and F2), one with recently familiarized odor (N1), and one with never encountered novel-odor from another donor rat (N2) following the same procedure outlined for the habituation phase. Exploration times for each bead were recorded. Data are presented as the percentage of exploration time on N2 relative to the total amount of exploration time spent on all beads. The focus of the test is to assess whether the test subject can identify the novelty of N2 by retaining the overnight memory for the N1 bead. Thus, a higher percentage of exploration time on N2 reflects better memory.

The social interaction test evaluates cognitive function in the form of sociability and social memory and novelty (Sato et al., 2012). Briefly, the social interaction test was performed in a three-chambered apparatus (Sato et al., 2012). After the testing rat habituated to the apparatus, a social target rat (R1) was randomly placed in the left- or right-compartment (the sociability test phase). The time spent by the rat exploring the R1 was recorded. The testing rat was then introduced to R1 rat and to a novel social target-never met before rat (R2) by randomly placing R1 and R2 in the left- or right-compartments (the social memory/novelty test phase). The times spent with R1 and R2 were recorded. The social memory/novelty data are presented as the percentage of time spent with R2 relative to the total amount of time spent with R1 and R2.

The MWM test is a widely used behavioral test for hippocampal-dependent spatial learning and memory (Choi et al., 2006). Rats were subjected to daily trial for five consecutive days starting at 4M after NTM-STZ injection. Briefly, on each testing day, the rat was placed in to a circular water tank (140 cm in diameter) with a transparent platform (15 cm in diameter) submerged 1.5 cm below the water at a random location within the Northeast (correct) quadrant of the tank. The rat was placed from four starting points (north, south, east, and west) in random order, and allowed to swim for a maximum of 90 s. The latency to find the hidden platform and the time spent within the correct quadrant were recorded. Data are presented as the latency to find the hidden platform (seconds) and the percentage of time spent within the correct quadrant relative to the total amount of time spent in the tank.

### Immunohistochemistry

Immunofluorescent staining for brain tissue (*n* = 8/group) and culture cells were performed according to our published protocols (Zhang et al., 2016). For each staining, four coronal sections (8 μm in thickness) spaced as 50–100 μm intervals, at the levels of lateral ventricle (bregma –0.4 to –1.4 mm) and dorsal hippocampus (bregma –2.8 to –3.8) per rat were used. The following primary antibodies were used: mouse anti endothelial barrier antigen (EBA, 836804, 1:1000, Biolegend), goat anti fibrin/fibrinogen (YBGMFBG, 1:1000, Accurate Chem.), mouse anti BrdU (M0744, 1:100, Dako), goat anti doublecortin (DCX, 1:100, sc-8066, Santa Cruz), mouse anti-Nestin (556309, 1:100, BD Biosciences), and mouse anti-β-III tubulin (TuJ-1, 801202, 1:1000, Biolegend). For quantification of vascular damage, the numbers of EBA positive vessels with fibrin/fibrinogen immunoreactivity within hippocampus were counted and are presented as the density of immunoreactive vessels relative to the scan area (mm^2^) determined with an MCID image analysis system (Imaging Research). For quantitative analysis of cell proliferation, the numbers of BrdU positive cells were counted throughout the subventricular zone (SVZ) of the lateral ventricle and granule cell layer of dentate gyrus (DG). Data are presented as the density of immunoreactive cells relative to the area of the SVZ and DG. For quantification of neuroblasts, the numbers of DCX positive cells in the DG were counted and presented as the density of immunoreactive cells relative to the DG area. Moreover, the number and the total length of branches emerging from the soma of DCX positive cells in the DG were measured. The DCX immunoreactive areas within the SVZ were measured and presented as a percentage of positive immunoreactive area relative to the area of SVZ. The percentage of BrdU cells double-labeled for DCX and Nestin within the SVZ were measured.

### Quantification of mature microRNAs and proteins

Subventricular zone and DG tissues were manually collected from frozen coronal brain sections (*n* = 6/group), as previously described (Guo et al., 2012). Total RNAs were isolated using the miRNeasy mini Kit (Qiagen), followed by reverse transcription. Individual RT and TaqMan real-time PCR reactions of miR-1 and –146a were performed on an ABI 7000 instrument (Applied Biosystems). Each TaqMan assay was done in triplicate for each sample tested. Relative quantities were calculated using the 2-ΔΔCT method with U6 small nuclear RNA TaqMan miRNA control assay (Applied Biosystems) as the endogenous control. Ingenuity Pathway Analysis (IPA, Qiagen) was used to generate the microRNA and target gene interaction network. IPA network analysis revealed that miR-1 and –146a can directly and/or indirectly target genes of thrombospondin 1 (TSP1) and myeloid differentiation primary response gene 88 (MYD88). Protein levels of MyD88 and TSP1 in isolated SVZ and DG tissues were measured by Western blot. The following primary antibodies were used: MyD88 (PA5-19918, 1:1,000, Invitrogen), TSP1 (37879s, 1:1,000, Cell Signaling Technology), and β-actin (1:4,000, Abcam). Signal bands were visualized by the ECL system (Amersham). The relative densities among the blot lanes were analyzed using the MCID system (Imaging Research).

### Cerebral endothelial cell-small extracellular vesicles labeling and *in vivo* tracking

To examine the brain distribution of intravenously administered CEC-sEVs, N-CEC-sEVs carrying CD63-GFP (N-CEC-sEVs-GFP) were isolated from CECs transfected with a plasmid carrying pEGFP-CD63 vector (Addgene) according to our previously published protocols (Zhang et al., 2021). Normal adult rats (*n* = 4) were subjected to intravenous injection of N-CEC-sEVs-GFP (1 × 10^11^ particle/injection) *via* a tail vein. Brain tissues were collected at 4 h after administration of N-CEC-sEVs-GFP. To examine the internalization of N-CEC-sEVs-GFP at the ultra-structural level, the lateral ventricle tissues were subjected to immunogold staining in which we employed streptavidin-gold nanoparticle (10 nm) conjugate (25269, EMS) and an anti-rabbit monoclonal antibody against GFP (G10362, ThermoFisher). TEM analysis was performed according to our published protocols (Zhang et al., 2021).

### Cerebral endothelial cell function assays

A sEV uptake assay was used to evaluate the internalization of exogenous sEVs by CECs. Briefly, N-CEC-sEVs or DM-CEC-sEVs were fluorescent-labeled with the ExoGlow Protein EV labeling kit (BSI# EXOGP300A-1) according to the manufacturer’s instruction. Primary CECs isolated from healthy young adult rats were cultured with fluorescent-labeled N-CEC-sEVs and DM-CEC-sEVs (3 × 10^7^ particles/ml) for 1 h, respectively. To further examine whether endocytosis pathways mediate the cellular uptake of sEVs, CECs pretreated with Pitstop-2 (clathrin inhibitor, 20 μM) and Nystatin (caveolin inhibitor, 20 μM) for 24 h were cultured with fluorescent-labeled sEVs (3 × 10^7^ particles/ml). The fluorescent signal in CECs were determined with an MCID image analysis system (Imaging Research).

To investigate whether sEVs modulate the barrier function of CECs, we harvested CECs from aged-DM rats (DM-CECs) 2M after NTM-STZ injection and age-matched non-DM rats (N-CECs). Endothelial monolayer barrier assay were performed according to our published methods^33^. Briefly, CECs harvested non-DM rats (N-CECs) and DM rats (DM-CECs, passage 3 to 4) were cultured for 7 days at a density of 5 × 10^4^ per well and were then seeded as monolayer onto the inner chamber of *trans*-well insert (Corning) in the presence or absence of DM-CEC-sEVs or N-CEC-sEVs (3 × 10^7^ particles/ml) for 48 h, respectively. Fluorescent-conjugated dextran (0.5 mg/mL, 70 kDa, D1830; Thermo Fischer Scientific) was then added to the top of the *trans*-wells for 30 min. Fluorescent signals in the bottom medium were measured using a plate reader at excitation and emission wavelengths of 595 and 615 nm, respectively. *Trans*-endothelial permeability was calculated as % signals = (optical density experimental-optical density vehicle)/optical density vehicle × 100. To examine whether blocking sEV uptake abolishes its effects on endothelial cell barrier function, CECs pretreated with endocytosis inhibitors were cultured in the presence and absence of DM-CEC-sEVs or N-CEC-sEVs. All data were obtained from 3 individual experiments.

### Subventricular zone cell culture and function assays

In the present study, the term of NSCs was used which includes neural progenitor and stem cells. In rodent brain, NSCs are present in the SVZ of the lateral ventricle and in the subgranular zone of the DG, and these cells generate new neurons throughout life (Zhang et al., 2004; Deng et al., 2009). To examine whether CEC derived sEVs modulate NSC function, the SVZ were dissociated from aged-DM rats (15 months of age, after 2 months of DM) and age-matched non-DM rats, as previously reported (Zhang et al., 2004). The cells were cultured in the medium containing 20 ng/ml basic fibroblast growth factor and epidermal growth factor at a cell density of 1 × 10^4^ cells/ml. To test the effects of DM-CEC-sEVs on NSCs harvested from healthy non-DM rats, SVZ cells from age-matched non-DM rats were cultured with and without DM-CEC-sEVs (3 × 10^7^ particles/ml). To test whether N-CEC-sEVs ameliorate cell proliferation and neuronal differentiation of NSCs harvested from aged-DM rats, SVZ cells from aged-DM rats were cultured with and without CEC-sEVs (3 × 10^7^ particles/ml). The number and size of neurospheres were measured after 7 day culture. To examine the effects of CECs derived sEVs on cell proliferation and neuronal differentiation, neurosphere cells were plated onto laminin-coated glass coverslips for differentiation assay according to our previously published protocols (Zhang et al., 2004). For cultured cells, BrdU and TuJ1 positive cells as well as total cells identified by 4’,6-diamidino-2-phenylindole nuclei counterstain were counted and presented as the percentage of each cell type in the total cells numbers.

### Statistical analysis

Repeated measure of analysis (ANCOVA) was performed to study the effects of CEC-sEV treatment on odor test, social chamber test, and MWM over time, respectively, and analysis considered treatment-by-time interaction. A significant time by treatment interaction (*p*-value < 0.05) indicated the treatment effects are dependent on time. One-way ANOVA with *post hoc* Bonferroni tests was used for multiple groups comparisons. Two-sample *t*-test was used for two group comparisons. Data are presented as mean ± standard error. A *p*-value < 0.05 was considered significant.

## Results

### Diabetes mellitus-cerebral endothelial cell-small extracellular vesicles reduce neurogenesis

**FIGURE 1:**
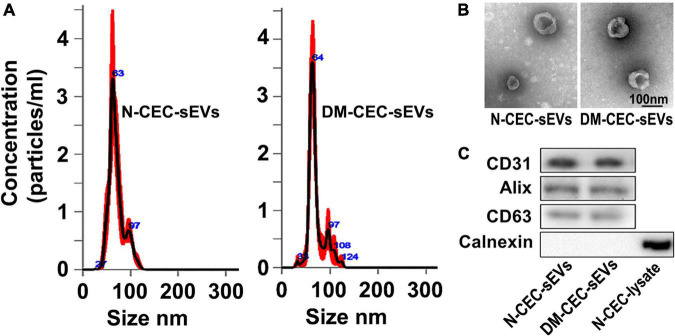
Characterization of CEC-sEVs. NTA **(A)** shows size and distribution of N-CEC-sEVs and DM-CEC-sEVs. TEM image **(B)** shows the cup-shaped structures of N-CEC-sEVs and DM-CEC-sEVs. Western blot images **(C)** show sEV membrane protein markers, but not intracellular protein calnexin.

To examine the effect of DM-CEC-sEVs on neurogenesis, we performed *ex vivo* experiments. CEC-sEVs were isolated from the medium of cultured primary CECs harvested from normal healthy young adult (2 to 3 months, N-CEC-sEVs) and aged-DM (15 month of age, 2M after NTM-STZ injection, DM-CEC-sEVs) male rats. Using multiple methods including NTA, TEM, and Western blot analysis, we found that N-CEC-sEVs and DM-CEC-sEVs had an average particle size of 70.5 ± 1.1 (N-CEC-sEVs) and 70.8 ± 1.4 (DM-CEC-sEVs), cup-shaped morphology, and exhibited sEV membrane marker proteins, but not intracellular protein calnexin, respectively, (Figure 1), which are consistent with the Minimal Information for Studies of Extracellular Vesicles 2018 (MISEV2018) report (Théry et al., 2018). There were no significant differences of the particle size, morphology, and protein component between N-CEC-sEVs and DM-CEC-sEVs.

**FIGURE 2:**
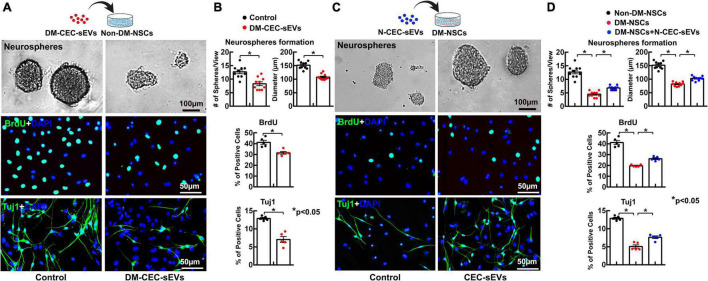
The effects of DM-CEC-sEVs and N-CEC-sEVs on NSCs. The schematic outlines the experimental protocol for the effect of DM-CEC-sEVs on healthy non-DM-NSCs and the representative images of non-DM-NSCs culture **(A)**. Quantitative data show DM-CEC-sEVs reduced the number and size of neurospheres, BrdU (green) positive cells, and Tuj1 (green) positive cells **(B)**. The schematic outlines the experimental protocol for the effect of N-CEC-sEVs on DM-NSCs and the representative images of DM-NSCs culture **(C)**. Quantitative data show N-CEC-sEVs increased number and size of neurospheres, BrdU positive cells, and Tuj1 positive cells **(D)**. *p < 0.05 versus indicated groups.

We then harvested NSCs from DM rats and age matched non-DM rats. Compared to NSCs harvested from non-DM rats, NSCs from DM rats showed significant reduction of neurosphere formation, cell proliferation, and neuronal differentiation (Figures 2A,C,D), which is consistent with published studies (Bonds et al., 2020). Treatment of NSCs harvested from DM rats with N-CEC-sEVs significantly increased neurogenesis (Figures 2C,D), whereas treatment of NSCs harvested from non-DM rats with DM-CEC-sEVs robustly suppressed neurogenesis (Figures 2A,B). These *ex vivo* data indicate that sEVs derived from dysfunctional CECs inhibit neurogenesis, whereas sEVs derived from healthy CECs alleviate DM-impaired neurogenesis.

### Treatment of aged-diabetes mellitus rats with N-cerebral endothelial cell-small extracellular vesicles enhances neurogenesis and improves cognitive function

**FIGURE 3:**
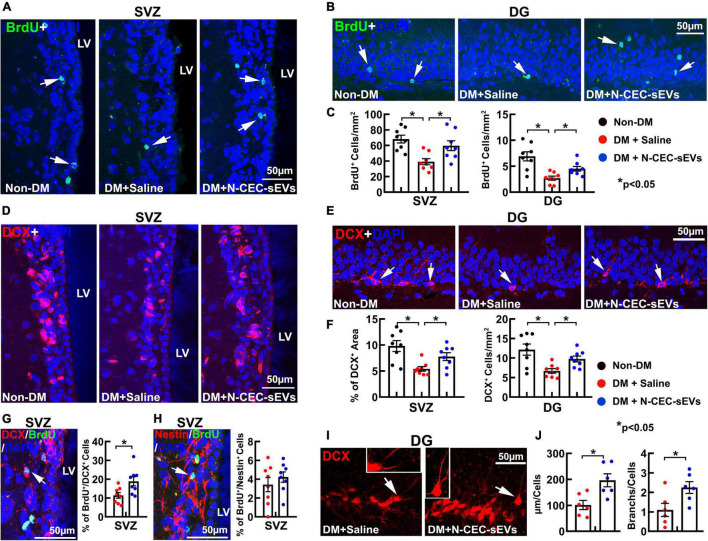
N-CEC-sEV treatment promotes neurogenesis. Representative microscopic images **(A,B)** and quantitative data **(C)** of the BrdU positive cells (green, arrow) in the SVZ **(A)** and the DG **(B)** of age-matched non-DM rats and aged-DM rats treated with saline and N-CEC-sEVs. Representative microscopic images **(D,E)** and quantitative data **(F)** show DCX^+^ neuroblasts (red) in the SVZ **(D)** and the DG **(E)**. Representative microscopic images of BrdU^+^ cells double labeled with DCX (**G**, arrow) and Nestin cells (**H**, arrow) in the SVZ of aged-DM rats treated with N-CEC-sEVs. Bar graphs in **(G,H)** are the quantitative data of BrdU^+^/DCX^+^ and BrdU^+^/Nestin^+^ double labeled cells in the SVZ of aged-DM treated with saline and N-CEC-sEVs. Representative confocal microscopic images of DCX^+^ neuroblasts (red) in the DG **(I)** of aged-DM rats. Inserts in **I** show a DCX^+^ neuroblast (arrow in Saline) with short processes, whereas a DCX^+^ neuroblast (arrow in N-CEC-sEVs) with long and branched processes. Bar graphs in **(J)** show N-CEC-sEVs increased branch number and length of DCX^+^ neuroblast. LV: lateral ventricle. *p < 0.05 versus indicated groups.

To extend aforementioned *ex vivo* finding, DM rats at age of 15 months (2M after NTM-STZ injection) were treated with N-CEC-sEVs (1 × 10^11^ particles/injection) two times a week for four consecutive weeks *via* a tail vein. To examine the effect of N-CEC-sEVs on neurogenesis, we employed a BrdU chasing approach that is a standard method to study neurogenesis (Wojtowicz and Kee, 2006), in which BrdU was given for seven consecutive days to the rat at age of 15 months while BrdU immunoreactive cells were examined 2 months after termination of BrdU treatment. Compared to age matched non-DM rats, DM rats had significant reduction of DCX+ neuroblasts and BrdU+ cells in the DG and the SVZ (Figures 3A–F), which is consistent with our previous findings (Zhang et al., 2016). Treatment of DM rats with N-CEC-sEVs significantly increased the number of BrdU+ cells and the DCX+ neuroblasts in the DG and the SVZ compared to saline treated DM rats (Figures 3A–F). Moreover, N-CEC-sEV treatment significantly increased BrdU+/DCX+ double labeled cells but not the BrdU+/Nestin+ cells in the SVZ compared to saline treatment (Figures 3G,H). Confocal microscopy analysis of DCX immunoreactive cells in the DG revealed that the N-CEC-sEV treatment significantly increased branch number and length of DCX^+^ neuroblasts compared to DCX+ neuroblasts in saline treated DM rats (Figures 3I,J), suggesting that DCX^+^ neuroblasts acquire a more mature phenotype after the N-CEC-sEV treatment. Collectively, these data indicate that N-CEC-sEV treatment enhances neurogenesis in DM rats.

**FIGURE 4:**
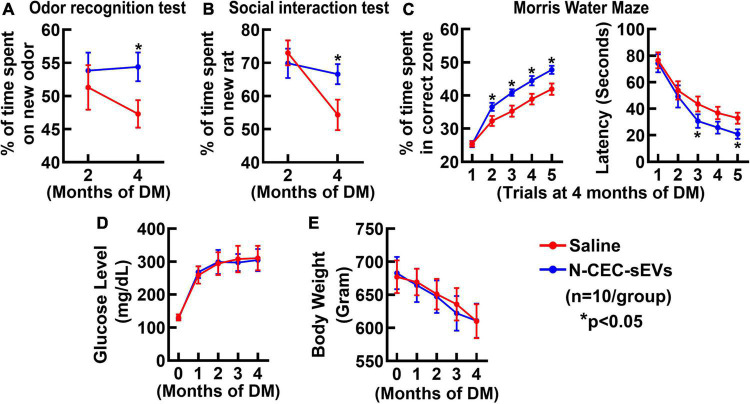
N-CEC-sEV treatment reduces cognitive deficits in aged DM rats. Odor recognition test **(A)**, social interaction test **(B)**, Morris water maze **(C)**, blood glucose level **(D)**, and body weight **(E)** in aged-DM rats treated with saline and N-CEC-sEVs. *P < 0.05 vs. the saline group. *N* = 10/group.

Neurogenesis contributes to cognitive function (van Praag et al., 2002; Deng et al., 2009). An array of behavioral tests was employed to examine cognitive function, which included an odor recognition test to evaluate the social recognition memory, a social interaction test to assess social memory and novelty, and a MWM test to specifically detect hippocampal-dependent spatial learning and memory (Levy et al., 2004; Choi et al., 2006; Sato et al., 2012). Compared to age matched non-DM rats, DM rats treated with saline spent significantly less time to explore a novel odor object and to interact with a new rat (Figures 4A,B). In contrast, DM rats treated with N-CEC-sEVs spent significantly more time (*p* < 0.05) on a novel object and to interact with a new rat compared to DM rats treated with saline. In addition, the MWM test revealed that DM rats treated with N-CEC-sEVs significantly increased their percent time in the correct quadrant and the escape latency compared to DM rats treated with saline (Figure 4C). These data indicate that the N-CEC-sEV treatment improves cognitive function in DM rats. The N-CEC-sEV treatment did not significantly alter blood glucose levels and body weight in DM rats (Figures 4D,E).

### Treatment of aged-diabetes mellitus rats with N-cerebral endothelial cell-small extracellular vesicles reduces diabetes mellitus damaged cerebral vasculature

**FIGURE 5:**
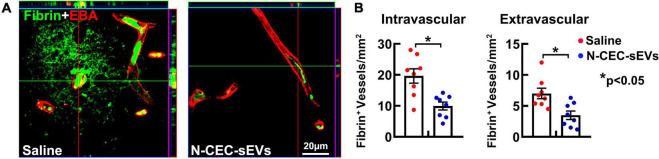
N-CEC-sEV treatment reduces cerebral vascular damage. Representative confocal microscopic images **(A)** and quantitative data **(B)** of fibrin/fibrinogen immunoreactivity (green) in the hippocampus of aged-DM rats treated with saline or N-CEC-sEVs. *p < 0.05 versus indicated groups.

We previously demonstrated that DM elicits cerebrovascular disruption characterized by microvascular thrombosis and blood brain barrier (BBB) leakage mainly localized to the hippocampus (Zhang et al., 2016). Thus, in addition to neurogenesis, we examined the effect of N-CEC-sEVs on DM-induced vascular damage. Treatment of DM rats with N-CEC-sEVs significantly reduced vascular thrombosis and BBB leakage as indicated by the decreased number of vessels with intra and extravascular fibrin deposition, respectively, in the hippocampi compared to DM rats treated with saline (Figure 5).

**FIGURE 6:**
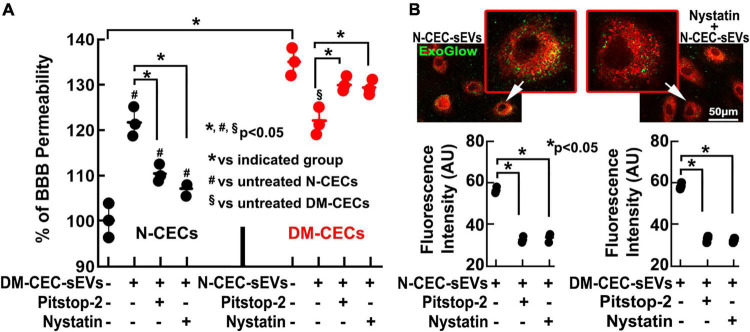
DM-CEC-sEVs increase healthy CEC permeability, whereas N-CEC-sEVs reduce DM-augmented permeability. Blockage of clathrin- and caveolin-pathways abolishes the effects of sEVs on CEC permeability. Quantitative data **(A)** of endothelial monolayer barrier permeability assay in N-CECs with and without DM-CEC-sEVs, and in DM-CECs with and without N-CEC-sEVs in the presence and absence of endocytosis inhibitors. Representative images and quantitative data **(B)** of sEVs internalization by N-CECs in the presence and absence of endocytosis inhibitors. *p < 0.05 versus indicated groups.

Using a cerebral endothelial permeability assay, we then examined a direct effect of N-CEC-sEVs on CECs. Compared to primary CECs harvested from age matched non-DM rats, CECs from DM rats showed significant leakage. However, addition of N-CEC-sEVs to CECs harvested from DM rats robustly reduced endothelial cell leakage (Figure 6A). Together, these *in vivo* and *ex vivo* data indicate that N-CEC-sEVs can reduce DM-induced cerebral vascular damage.

The clathrin and caveolin signaling pathways mediate endocytosis of extracellular vesicles (Mulcahy et al., 2014). We thus, examined whether these two pathways are involved in the up-take of sEVs by CECs. After incubation of CECs harvested from non-DM rats with fluorescently labeled CEC-sEVs derived from non-DM rats (N-DM-CEC-sEVs) or from DM rats (DM-CEC-sEVs), fluorescent signals were detected within the cytosol of CECs, indicating that endothelial cells internalize sEVs (Figure 6B). There were no significant differences of EV uptake between N-DM-CEC-sEVs and DM-CEC-sEVs (Figure 6B). Pretreatment of endothelial cells with pitstop2, a cell-permeable clathrin inhibitor, or nystatin that inhibits caveolin-dependent endocytosis, significantly reduced uptake of N-DM-CEC-sEVs and DM-CEC-sEVs (Figure 6B), suggesting both signaling pathways are involved in cerebral endothelial cell endocytosis of CEC-sEVs. Moreover, inhibition of endocytosis by the two inhibitors significantly reduced DM-CEC-sEVs increased endothelial cell permeability (Figure 6A), indicating that impairment of endothelial cell permeability by DM-CEC-sEVs is specific.

### Intravenously administered N-cerebral endothelial cell-small extracellular vesicles are internalized by neural stem cells and cerebral endothelial cells

**FIGURE 7:**
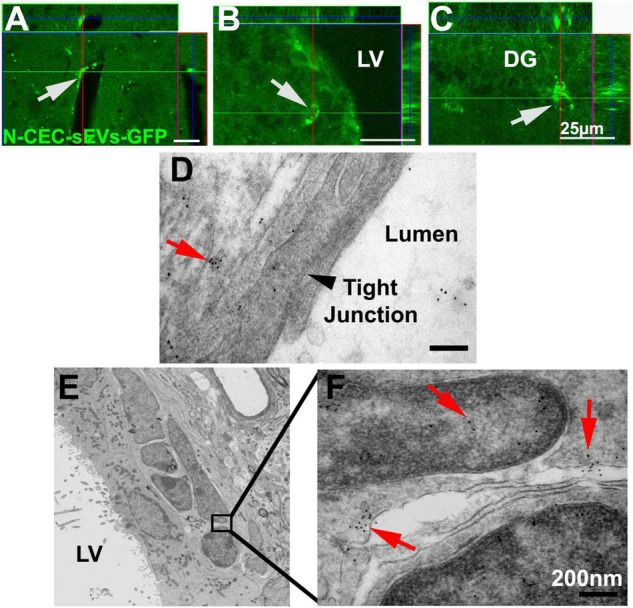
N-CEC-sEVs intravenously administered cross the BBB and are internalized by NSCs. Representative confocal microscopic images show GFP signals (green, arrows) in cells lining cerebral vessels **(A)** and cells within the SVZ **(B)** and DG of hippocampus **(C)** after IV injection of N-CEC-sEVs-GFP. Representative TEM images **(D–F)** show GFP positive immunogold particles (red arrows) within endothelial cells that lining the cerebral blood vessel luminal surface **(D)**, where tight junction was intact (black arrowhead in **D**). GFP positive immunogold particles (10 nm, **E,F**) were within sEVs (red arrows) localized to cytoplasm and nucleus of SVZ cells. LV, lateral ventricle. Bars = 25 μm in **(A–C)**. Bars = 200 nm in **(D,F)**.

We previously demonstrated that intravenously administered CEC-sEVs cross the BBB (Li et al., 2021). However, whether systemic administration of N-CEC-sEVs reach to NSCs has not been investigated. To track N-CEC-sEV in neurogenic regions, N-CEC-sEVs carrying CD63-GFP (N-CEC-sEVs-GFP) were intravenously administered to non-DM adult rats. The rats were sacrificed 4 h after the treatment and their brains were processed for analysis. Confocal microscopic analysis (Figures 7A–C) showed that GFP signals were detected within NSCs in the neurogenic regions of the SVZ and the subgranular zone of DG (Figures 7B,C). Ultrastructural analysis (Figures 7D–F) revealed that GFP-immunogold positive particles were localized to cytoplasm and nucleus of NSCs in the SVZ (Figures 7E,F), indicating that NSCs internalize CEC-sEVs. Consistent with our previous findings (Li et al., 2021), GFP signals (Figure 7A) and GFP-immunogold positive particles (Figure 7D) were also detected in cerebral endothelial cells.

### Treatment of diabetes mellitus-rats with N-cerebral endothelial cell-small extracellular vesicles augments miR-1 and –146a and reduces their target proteins in neurogenic regions of the dentate gyrus and the subventricular zone

**FIGURE 8:**
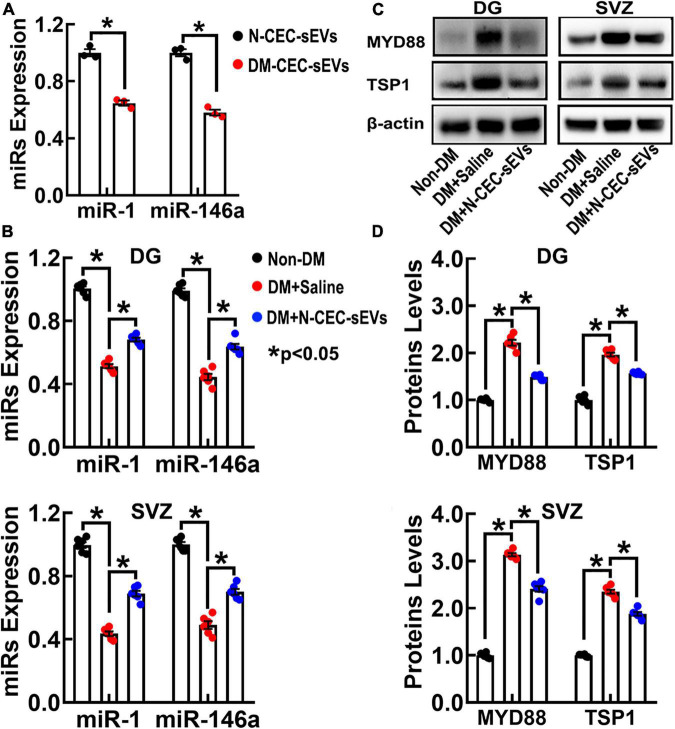
N-CEC-sEV treatment elevates miR-1 and –146a, and reduces MYD88 and TSP1 expression in DG and SVZ tissues isolated from aged-DM rats. Quantitative RT-PCR data show miR-1 and –146a levels in N-CEC-sEVs and DM-CEC-sEVs **(A)** and in DG and SVZ tissues **(B)** isolated from age-matched non-DM rats, and aged-DM rats treated with saline and N-CEC-sEVs. Representative Western blots **(C)** and quantitative data **(D)** of MYD88 and TSP1 levels in the DG and the SVZ. Data are presented as fold change from the non-DM group. *p < 0.05 versus indicated groups.

We and others have demonstrated that miRNAs regulate neurogenesis through modulating their target genes (Chamorro-Jorganes et al., 2011). Small EVs impact recipient cell biological function by delivering their cargo materials including miRNAs (O’Brien et al., 2020; Zhang et al., 2021). Using qRT-PCR analysis of N-CEC-sEVs, we found that miR-1 and –146a were 2.2 and 1.9-fold higher than DM-CEC-sEVs (Figure 8A). Next, we measured these two miRNA levels in isolated DG and SVZ tissues. Compared to age matched non-DM rats, DM dramatically decreased miR-1 and –146a levels, whereas the N-CEC-sEV treatment significantly increased miR-1 and –146a levels compared to the tissues isolated from saline treated DM rats (Figure 8B). Bioinformatics analysis revealed that miR-1 and –146a can directly and/or indirectly target genes of MYD88 and TSP1. MYD88 related toll-like receptor (TLR) signaling is involved in vascular dysfunction, while TLR regulate adult neurogenesis (Rolls et al., 2007; Okun et al., 2011). Western blot analysis of DG and SVZ tissues showed that compared to age-matched non-DM rats, aged-DM rats exhibit significantly increased MYD88 and TSP1 levels. In contrast, the N-CEC-sEV treatment significantly reduced MYD88 and TSP1 levels compared to the saline treatment (Figures 8C,D). An inverse relationship of the miR-1 and –146a and the protein levels of MYD88 and TSP1 in the DG and SVZ suggests that alterations of this network by CEC-sEVs likely contribute to the therapeutic effect of CEC-sEVs on DM rats.

## Discussion

Although studies have demonstrated that DM induces cerebral vascular dysfunction and impairment of neurogenesis, the mechanistic interactions between the vascular dysfunction and reduced neurogenesis under DM remains to be investigated. The present study demonstrated that (1) DM-CEC-sEVs impair neurogenesis and induce cerebral endothelial dysfunction, (2) N-CEC-sEVs robustly improve DM-impaired cognitive function with augmentation of neurogenesis and amelioration of dysfunctional cerebral vasculature in aged DM rats, and (3) increases of miR-1 and –146a and reductions of their target genes such as MyD-88 and TSP1 may contribute to the therapeutic effect of CEC-sEVs.

Endothelial cell dysfunction contributes to cerebrovascular disease that is a major complication of DM (Wang et al., 2018). Experimental studies have demonstrated that DM causes cerebral vascular dysfunction, impairment of neurogenesis, and cognitive decline, which resemble the neuropathological manifestation of DM patients (Stranahan et al., 2008; Zhang et al., 2016). Small EVs play an important role in cell to cell communication by transferring their cargo biological materials, leading to changes in recipient cell biological function under healthy and disease conditions (Valadi et al., 2007; Kourembanas, 2015). However, there are limited studies to investigate the role of sEVs in mediating dysfunctional CECs and neurogenesis impairment under DM. The present study provides the first evidence that DM-CEC-sEVs suppress healthy NSC proliferation and differentiation and induce cerebral endothelial cell dysfunction. Our *ex vivo* and *in vivo* data demonstrated that N-CEC-sEVs are internalized by CECs and NSCs, while the clathrin- and caveolin-dependent endocytosis signaling pathways mediate uptake of CEC-sEVs by recipient cells. Inhibitions of either one of these two pathways substantially reduced DM-CEC-sEV-impaired vascular function, which is consistent with studies in the literature showing that these two endocytosis pathways are critical in mediating EV endocytosis (Mulcahy et al., 2014). The present findings are in line with emerging evidence that cerebral vascular dysfunction triggers neuronal damage and consequently leads to impairment of cognitive function (Gorelick et al., 2011). Furthermore, our data indicate that DM-CEC-sEVs play an important role in processes of cerebral vascular dysfunction and impairments of neurogenesis. Recent studies have shown that blood plasma sEVs derived from diabetic patients induce dysfunction of immune cells and human aortic endothelial cells (Freeman et al., 2018; Wu et al., 2020). Thus, the present findings have translational values to provide a possible mechanism by which sEVs provide critical linkage between DM-induced cerebral vascular dysfunction and neurogenesis impairment.

In contrast to DM-CEC-sEVs, N-CEC-sEVs showed a robust therapeutic effect on amelioration of DM-induced cognitive deficits with reducing cerebral vascular thrombosis and leakage and augmenting neurogenesis in aged DM rats. Clinical trials demonstrate that intensive glucose control in patients with T2DM does not improve cognitive function (Peñaherrera-Oviedo et al., 2015; Simó et al., 2017). The present study shows that N-CEC-sEVs did not significantly alter blood glucose levels and animal body weight, suggesting that the therapeutic effect of N-CEC-sEVs on aged DM rats is independent on changes of blood glucose levels. As confirmed at ultrastructural levels, intravenously administered N-CEC-sEVs crossed the BBB and were internalized by NSCs. *Ex vivo* experiments also demonstrated that N-CEC-sEVs internalized by DM-CECs and NSCs reversed DM-impaired cerebral endothelial cell function and neurogenesis. Importantly, experimental data demonstrate that neurogenesis is highly related to cognitive function (van Praag et al., 2002; Deng et al., 2009). Although adult neurogenesis in humans remains a controversial area of research (Moreno-Jiménez et al., 2021), recent studies showed that neurogenesis occurs in the hippocampus of adult neurologically healthy human subjects, whereas patients with Alzheimer’s disease have substantial reduction of hippocampal neurogenesis (Spalding et al., 2013; Moreno-Jiménez et al., 2019). Together, our data suggest that N-CEC-sEVs likely act on CECs and NSCs, particularly on enhancement of neurogenesis, to achieve the therapeutic effect on improvement of cognitive function. One should be noted that the N-CEC-sEV treatment was initiated in rats subjected to 2-month hyperglycemia. Additional experiments are warranted to investigate whether N-CEC-sEVs retain their therapeutic effect when the treatment is initiated in aged rats subjected to a long-term hyperglycemia.

Small EVs carry genetic materials including miRNAs, and transfer their cargo miRNAs to recipient cells to evoke changes of recipient cell biological function (Valadi et al., 2007). The present study showed that DM significantly reduced miR-1 and –146a in the DG and the SVZ, which is consistent with clinical and preclinical data showing reduction of that miR-1 and miR-146a levels in the circulation and cerebrospinal fluid of patients with diabetes (Baldeón et al., 2014; Quintana et al., 2017; Kokkinopoulou et al., 2019) and down-regulation of miR-1 in endothelial cells by hyperglycemia (Feng et al., 2014). miR-1 directly targets TSP1 that triggers vascular thrombotic formations (Bonnefoy and Hoylaerts, 2008). miR-146a targets TLR signaling including downstream MyD88 (Saba et al., 2014), whereas MyD88 deficiency in adult rodent increases hippocampal neurogenesis (Rolls et al., 2007; Okun et al., 2011). Our data show that N-CEC-sEVs are enriched with miR-1 and –146a and that the N-CEC-sEV treatment robustly increased miR-1 and –146a in the DG and the SVZ, which is inversely associated with the reductions of MYD88 and TSP1. We and others have previously demonstrated that CECs derived sEVs/exosomes express miR-146a, and upregulation of miR-146a reduces cerebral microvascular damage and promotes neurogenesis (Liu et al., 2017; Li et al., 2021). Following endocytosis, sEV cargo single-stranded miRNAs are loaded onto the recipient cell argonaute proteins on the endosomal membrane to regulate their target genes in recipient cells (Ghoshal et al., 2021). Compared to other nanoparticles, sEVs deliver their cargo RNAs to recipient cells at ∼100 fold efficiency (Murphy et al., 2021). Thus, N-CEC-sEVs could alter a network of miRNAs and their target genes in recipient CECs and NSCs, subsequently leading to reduction of DM induced vascular dysfunction and impairment of neurogenesis. *Further investigation* is warranted to validate the specific targets of miR-1 and –146a. Moreover, future casual studies to alter N-CEC-sEV cargo miRNAs and to investigate the roles of cargo proteins on the therapeutic effect of NCEC-sEVs could provide additional mechanistic information as to how N-CEC-sEVs reverse DM-induced cerebral vascular dysfunction and neurogenesis impairment.

In conclusion, the present study provides novel *ex vivo* and *in vivo* evidence that DM-CEC-sEVs induce cerebral vascular dysfunction and neurogenesis impairment, whereas N-CEC-sEVs have a therapeutic effect on improvement of cognitive function by ameliorating dysfunction of cerebral vessels and neurogenesis in aged male DM rats, respectively. Given the multifaceted pathophysiological nature of aging and DM, N-CEC-sEVs by acting on multiple cellular components that are important for cognitive function represent a novel strategy to treat aging and DM-induced cognitive dysfunction.

## Data Availability Statement

The raw data supporting the conclusions of this article will be made available by the authors, without undue reservation.

## Ethics statement

The animal study was reviewed and approved by Institution Animals Care and Use Committee of Henry Ford Hospital.

## Author contributions

LZ, ZZ, and MC contributed to conception and design of the study. ML performed the statistical analysis. LZ, CL, RH, HT, YZ, MZ, XL, BF, HL, AH, and AZ contributed to data acquisition, analysis, and interpretation. All authors contributed to manuscript revision, read, and approved the submitted version.

## Conflict of Interest

The authors declare that the research was conducted in the absence of any commercial or financial relationships that could be construed as a potential conflict of interest.

## Publisher’s Note

All claims expressed in this article are solely those of the authors and do not necessarily represent those of their affiliated organizations, or those of the publisher, the editors and the reviewers. Any product that may be evaluated in this article, or claim that may be made by its manufacturer, is not guaranteed or endorsed by the publisher.
